# Summer Diarrhœa

**Published:** 1899-07-08

**Authors:** 


### SUMMER DIARRHŒA.

Lecturing in New York on what he calls "Summer Complaint in Children," Dr. Louis Fischer describes the disease as arising from fermentive and putrefactive changes in the contents of the digestive canal, as the result of which, if vomiting be not quickly set up, foul-smelling diarrhœa will surely follow. In the treatment of this condition he says that the best plan is to aid nature by giving a large dose of castor oil. If this is thrown up the dose must be repeated. Calomel is also a good remedy. It stimulates the flow of the bile, acts as an intestinal antiseptic, and by its purgative action removes the cause of the disease. Irrigation is of great service, the stomach being washed out with warm saline solution until the contents flow away clear, and the same thing being done to the lower bowel. The intense thirst which is met with in this disease is much relieved by the process; in fact, thirst is but a signal of a lack of fluid, and when, as happens sometimes, children are found to be in a state of collapse from excessive diarrhœa, the injection hypodermically of several pints of warm saline solution of the temperature of the blood is one of the most valuable means of restoring them. When there is thirst also a drink of boiled and cooled water, acidulated with hydrochloric or phosphoric acid, and sweetened with either glycerine or saccharine, is both useful and grateful to the little patient. As regards diet, which is more important than medicinal treatment, the first thing is to discontinue all those foods which were being given at the time the attack came on, and this applies especially to milk. In place of this, barley water, rice water, sago water, &c., may be given, but at somewhat longer intervals than the child is accustomed to, plain boiled water, or boiled water to which the white of a raw egg and some salt are added (albumen water) being given in the interval, and if the child is old enough an occasional few drops of the expressed juice of meat, made by broiling a steak over the fire, and expressing the juice in a lemon squeezer or meat press, may be added. Only after the infant's normal condition is restored should it return to its natural food—milk—and the greatest care should be taken to ensure that this is sterilised, or at any rate Pasteurised. Another point worth bearing in mind in the treatment of this very fatal disease is the great advantage which is often derived from change of air. It is a well ascertained fact (says Dr. Fischer) that a child suffering from summer complaint in the midst of a hot city will become almost constipated on being given a sea voyage.

